# Acute Respiratory Distress Syndrome in Patients with Cardiovascular Disease

**DOI:** 10.14797/mdcvj.1244

**Published:** 2023-08-01

**Authors:** Asma Zainab, Megan Gooch, Divina M. Tuazon

**Affiliations:** 1 Methodist DeBakey Heart & Vascular Center, Houston Methodist, Houston, Texas, US; 2 Weill Cornell Medical College, New York, US; 3 Houston Methodist, Houston, Texas, US

**Keywords:** ARDS, cardiovascular disease, right ventricular dysfunction, sepsis, pulmonary hypertension

## Abstract

Heart and lung interaction within the thoracic cavity is well known during inhalation and exhalation, both spontaneously and during mechanical ventilation. Disease and dysfunction of one organ affect the function of the other. A review of the cause-and-effect relationship between cardiovascular disease and acute respiratory distress syndrome (ARDS) is of significance, as the disease burden of both conditions has both a national and global impact on health care. This literature review examines the relationship between cardiovascular disease and ARDS over the past 25 years.

## Introduction

It is well established that cardiovascular disease is the leading cause of death in the United States (US),^1^ reportedly contributing to more than 870,000 deaths in 2019.^2^ Acute respiratory failure is a major reason for intensive care unit (ICU) admissions following cardiovascular surgery,^3,4^ and acute respiratory distress syndrome (ARDS) is one of the leading causes of acute respiratory failure among this group of patients.^5^ ARDS affects roughly 3 million people each year^5^ and has an incidence of 64.2 to 78.9 cases per 100,000 person-years. It contributes between 10% to 15% of ICU admissions,^6^ and 24% of these patients require mechanical ventilation.

Cardiovascular disease (CVD) carries a high disease burden in the US and globally, and patients with CVD who develop ARDS may have a mortality risk as high as 35% to 46%, depending on the severity. In addition, one in five cases of ARDs may be missed, adding to misdiagnosis and treatment and greater adverse outcomes.^7^

High mortality rates in both heart disease and ARDS increase the importance of understanding the relationship between the two diseases, especially since heart-lung interactions within the thoracic cavity are a well-known paradigm. This article reviews the cause-and-effect relationship between both conditions.

## Heart-lung Interactions During Spontaneous and Positive Pressure Ventilation

Heart-lung interactions occur continuously since they are both contained within the thoracic cavity. The pressure within the thoracic cage depends on lung inflation, changes in pleural pressure, and transpulmonary pressure. Within this pressure chamber, the heart is present as another pressure chamber, generating pressure during its systole-diastole cycle. Pressure changes during the respiratory cycle affect the pressure within the heart. Respiratory mechanics affect pulmonary circulation along with ventricular filling and ejection. During normal inspiration, the negative intrathoracic pressure leads to an increase in venous return but decreases ejection by the left ventricle, and the opposite occurs during expiration.

During positive pressure ventilation (PPV), increased positive end-expiratory pressure (PEEP) or tidal volumes increase pulmonary arterial resistance, leading to the impedance of right ventricular (RV) ejection and low cardiac output.^8^ Inflation of the lungs is associated with increased alveolar pressure and stretching, which reduces interstitial pressure and compression of pulmonary vessels in interstitial space, thus increasing transmural pressure on the vessel.^9^ Increased pulmonary vascular pressure contributes to low cardiac output due to an increase in RV afterload. Another factor is the ventricular interdependence secondary to the septum and shared pericardial space, so the diastolic pressure of the RV affects the diastolic filling of the left.^10^

## Cause-and-effect Relationship between CVD and ARDS

**Figure 1:**
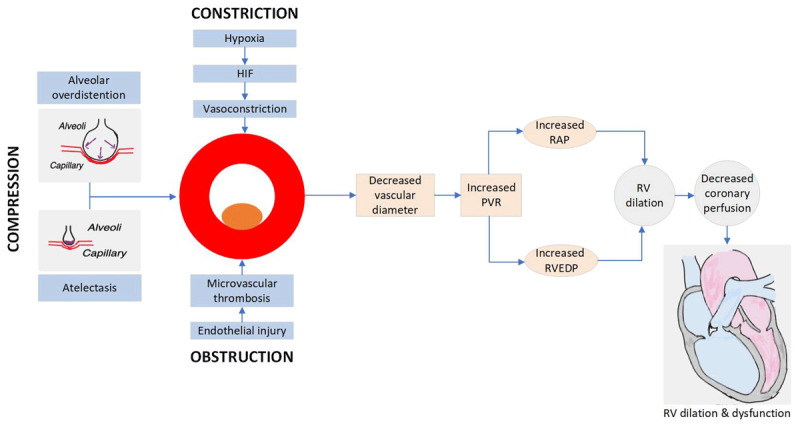
Pulmonary vascular dysfunction in acute respiratory distress syndrome. HIF: hypoxia inducible factor; RAP: right atrial pressure; RVEDP: right ventricular end diastolic pressure

ARDS may be due to direct lung injury or indirect injury due to nonpulmonary reasons. Regardless of the reason, endothelial and epithelial damage leads to diffuse alveolar damage and increased capillary permeability. The process involves neutrophil activation and migration, proinflammatory cytokine release, and activation of procoagulant molecules. Both the alveolar compartment and pulmonary circulation are affected (Figure 1).

Injury of the alveolar-capillary membrane barrier is worsened by mechanical ventilation, leading to spreading of the injury from the lungs to other organs. Strong evidence suggests that ARDS ultimately affects the heart. The relationship between CVD and ARDS (Table 1) is presented as three groups: ARDS secondary to cardiac etiology, ARDS leading to cardiac dysfunction, and ARDS and cardiomyopathy as a disease complex.

**Table 1:** Cause and effect relationship between cardiovascular disease and acute respiratory distress syndrome. RV: right ventricular


Cardiovascular disease as the cause: Cardiac arrest Post cardiotomy

Acute respiratory distress syndrome as the cause: Pulmonary hypertension and RV dysfunction Cardiac phenotypes

Cardiovascular disease and acute respiratory distress syndrome together Viral infections (eg, influenza, COVID-19) Severe sepsis and septic shock from any pathogen


## ARDS Secondary to Cardiac Etiology

### Post Cardiac Arrest ARDS

Studies show that at least 50% of patients who experience out-of-hospital cardiac arrest develop ARDS.^11^ These patients had higher mortality and longer ICU stays with more ventilator days. In another study on the incidence of in-hospital cardiac arrest, ARDS developed within 3 days of cardiac arrest in as many as 72% of patients. These patients had higher mortality and poorer neurologic outcomes compared with cardiac arrest patients who did not develop ARDS.^12^

ARDS after cardiac arrest may develop secondary to aspiration, lung contusion during chest compression, and ventilator-induced lung injury. A similar study stated that 41% of patients developed lung contusion after chest compressions.^13^

Ischemia and reperfusion injury after cardiac arrest lead to cytokine release and inflammation.^14^ The inflammatory response associated with endothelial injury, cytokine release, and complement activation is the hallmark of post-cardiac arrest syndrome and also associated with multiple organ injury, including ARDS.

### Post Cardiotomy ARDS

ARDS following cardiac surgery is rare, with an incidence of approximately 1.1% but with increased morbidity and mortality.^15^ Although significant progress has been achieved in the diagnosis and treatment of ARDS, it remains the leading cause of hypoxic respiratory failure in the postoperative period, with a mortality rate of almost 40% in the general population and 80% in patients undergoing cardiac surgery. The impact of ARDS in the cardiac surgery population is significant, affecting survival, in-hospital length of stay, and long-term physical and psychological morbidity. Every patient undergoing cardiac surgery can be considered at risk for ARDS. Therefore, it is crucial to identify those patients at higher risk as early as possible to adopt specific preventative and risk reduction strategies.^16^

### Diagnosis After Surgery

Diagnosing ARDS after cardiac surgery presents unique challenges because of the potential contribution of cardiac dysfunction. The development of ARDS is likely multifactorial, and the presence of preexisting cardiac dysfunction theoretically aggravates the degree of pulmonary edema in patients who develop ARDS.^17^ An accurate diagnosis of ARDS is critical because optimal treatment strategies—which include the use of high PEEP, low tidal volume (V_T_) ventilation, and permissive hypercapnia^18^—may conflict with treatment for cardiac surgical patients, specific patients with poor RV function, and patients with recent sternotomy. Furthermore, the increased risk of ARDS after cardiac surgery has traditionally been associated with the use of cardiopulmonary bypass (CPB), the need for blood product transfusions, large volume shifts, mechanical ventilation, and direct surgical insult.^16^

### Risk Factors

In patients who develop ARDS after cardiac surgery, research shows a quantitative relationship between CPB time and clinical outcomes, whereby long CPB duration results in poorer clinical outcomes.^19^ Techniques to reduce the time on CPB may be useful. However, eliminating CPB does not remove all potential stimuli of lung injury. Numerous risk factors are intrinsic to any cardiac operation, and some may preexist in the patient before surgery.^15^

Christensen et al. proposed the following as risk factors: postoperative low cardiac output, current smoking, left ventricular ejection fraction < 40%, hypovolemic hypotension, New York Heart Association class III and IV, hypertension, combined cardiac procedures, and emergency operation. Kaul and colleagues only found recent smoking, advanced chronic obstructive pulmonary disease, and emergency coronary artery bypass grafting to be associated with the development of ARDS. Milot et al. depicted previous cardiac surgery, shock, and multiple transfusions as predictors of ARDS. Kogan and colleagues identified previous cardiac surgery, complex cardiac surgery, and more than three transfusions with packed red blood cells. Age, liver cirrhosis, massive blood transfusions, and tricuspid valve replacement were recently reported as independent risk factors for ARDS in the subset of patients undergoing valvular surgery.^16^

### Risk Reduction

Cardiac anesthesiologists and intensivists believe that some key aspects may contribute to reducing the incidence of postoperative ARDS after cardiac surgery, such as optimization of mechanical ventilation (ie, use of low tidal volume, optimal PEEP, and lowest driving pressure), limitation of blood product transfusion (with restrictive hemoglobin target of 7 g/dL, reduction of hemodilution, and avoidance of fluid overload), and consideration of the hemodynamic effect of the mechanical ventilator on the RV function (ie, evaluation of RV function, establishment of adequate hemodynamic support, and avoidance of hypoxia, hypercarbia and acidosis).^17^

## ARDS as the Primary Cause of Cardiac Dysfunction

### Pulmonary Hypertension and RV Dysfunction

In normal circumstances, the three lung zones keep a balance between alveolar, arterial, and venous pressure. In injured lungs, however, the inflamed, collapsed, and fluid-filled alveoli lead to an imbalance. Along with that, the pressure drop in the pulmonary vasculature with PPV is greater than in nonventilated breath. Cyclic interruption of pulmonary flow due to alveolar distention or high peak airway pressure leads to microvascular injury, which in turn leads to pulmonary edema, high pulmonary vascular pressures, and eventually RV dysfunction.^8^

The mechanism of pulmonary hypertension (PH) in ARDS is multifactorial and includes hypoxia-related pulmonary vasoconstriction, endothelial dysfunction with activation of complement and release of cytokines leading to micro thrombosis, and alteration of the normal balance of mediators of vasodilation and vasoconstriction.^20,21^ Pulmonary hypertension in ARDS due to hypoxia and lung injury comes under WHO group 3 PH.^22^

Acute PH in the setting of ARDS leads to increased RV afterload. In mechanically ventilated patients, driving pressure > 18 cm H_2_O is considered a risk factor for RV injury.^23^ RV injury was noted to be present in 21% of ARDS patients in one meta-analysis, increasing overall and short-term mortality.^24^

### Cardiac Sub-phenotypes in ARDS

Interest in cardiovascular sub-phenotypes has recently increased. One study performed latent class analysis of > 800 patients for echocardiographic and hemodynamic parameters, and four cardiovascular sub-phenotypes were identified^25^: (1) Preserved RV: normal biventricular function; (2) RV dilation but preserved function: mild RV dilation and preserved cryptogenic organizing pneumonia (COP); (3) RV failure: RV dilation and dysfunction with low COP; and (4) hyperdynamic: high COP and LVEF.

At 78%, the mortality rate was highest among group 3, with RV failure. These patients had higher central venous pressure, low COP, and organ dysfunction. The hyperdynamic class presented with vasodilation, tachycardia, and high COP. This class was associated with the inflammatory state.

Another single-center study reviewed cardiovascular phenotypes in COVID-19 ARDS.^26^ The authors described three sub-phenotypes based on right ventricular function: (1) Preserved RV function or normal RV; (2) RV dysfunction with dilated RV but normal function; and (3) RV failure with dilated RV and severely impaired RV function. These sub-phenotypes have identified that the association of RV failure with ARDS leads to higher mortality.

## ARDS and Cardiomyopathy as Disease Complex

### Infectious ARDS and Cardiomyopathy (Direct Injury)

Infectious diseases are a major cause of morbidity and mortality worldwide, with ARDS and cardiomyopathy being two of the most severe complications. While ARDS and cardiomyopathy often occur independently, there are instances when they occur simultaneously.

Several infectious diseases have been linked to the development of ARDS and cardiomyopathy concurrently. Viral infections that commonly cause ARDS and cardiomyopathy include COVID-19, influenza, adenovirus, and coxsackievirus.^27,28,29,30^ Some bacterial infections that typically lead to both disease processes are *Streptococcus pneumoniae*^31^ and *Staphylococcus aureus*.^32^ In general, ARDS and cardiomyopathy may develop as a result of sepsis from bacterial or viral infections.

### Direct Viral or Bacterial Invasion of the Heart and Lungs

In some cases, the infectious agent directly invades the heart tissue, leading to myocarditis and subsequent cardiomyopathy. This can occur with viruses as well as bacteria.^29,30,31,32^ The production of toxic shock toxin 1, an exotoxin, by staphylococcus aureus in toxic shock syndrome may lead to cardiac dysfunction.^32^ The direct invasion of the cardiac tissue by infectious organisms also may lead to the release of cytokines and chemokines, which can promote an inflammatory response and contribute to the development of ARDS.^33^ Influenza and COVID-19 can directly invade the respiratory system. Studies have demonstrated that both viruses can cause myocarditis and lead to cardiomyopathy through direct invasion of cardiomyocytes.^34,35^

The treatment of ARDS and cardiomyopathy depends on the underlying infectious disease. Influenza and COVID-19 may be treated with antiviral medications, such as oseltamivir and remdesivir, respectively, and supportive care.^16,17^ Bacterial sepsis is treated with antibiotics and supportive care. Patients with ARDS and cardiomyopathy may require life support measures such as mechanical ventilation, extracorporeal membrane oxygenation, and inotropic support.

### Severe Sepsis and Septic Shock with Multiorgan Dysfunction (Indirect Injury)

Severe sepsis is defined as sepsis with organ dysfunction, and when hemodynamic instability is present, it leads to septic shock. Both conditions are associated with in-hospital mortality as high as 38.4% and high costs.^36^

Sepsis in any part of the body can lead to widespread response secondary to endothelial injury, cytokine release, circulating chemokines, and acute-phase reactants as well as activation and infiltration of neutrophils with local and systemic neutrophil extracellular traps formation.

### Lung and Cardiac Dysfunction as Part of Multiorgan Failure in Severe Sepsis

Cardiac dysfunction is seen as impaired contractility, reduced cardiac index, and low ejection fraction.^37^ It is a well-known fact that inflammatory cytokines such as interleukin-1 (IL-1), IL-6, IL-8, and tumor necrosis factor-alpha (TNF-α) lead to lung inflammation and myocardial depression.^37,38^

#### Systemic Inflammatory Response

The development of ARDS and cardiomyopathy may be due to a systemic inflammatory response caused by an infectious agent. Cytokine storm is a systemic inflammatory response that can occur in response to a viral or bacterial infection. This response can lead to a surge of cytokines, including IL-6, IL-1, and TNF-α, which can lead to widespread tissue damage.^39^ Cardiac and pulmonary involvement can lead to cardiomyopathy and ARDS, respectively. COVID-19 has been associated with cytokine storm, which contributes to the development of severe ARDS and cardiac disease, particularly left ventricle dysfunction.^40,41^

#### Oxidative Stress

Another proposed mechanism of the co-occurrence of ARDS and cardiomyopathy is oxidative stress. The release of reactive oxygen species (ROS) by the immune system can lead to oxidative damage of the heart tissue, contributing to the development of cardiomyopathy.^42^ Oxidative stress can also cause damage to the pulmonary tissue, leading to the development of ARDS.^43^

### Immune-mediated Injury

Immune-mediated injury has been proposed as a mechanism for the development of ARDS and cardiomyopathy in infectious diseases. This response occurs when the immune system mistakenly attacks healthy tissues, including the heart and lungs. A recent study demonstrated that a subset of patients with severe COVID-19 developed autoantibodies that targeted multiple organs, including the heart and lungs. This response may play a role in the development of myocarditis and ARDS.^44^

### Microvascular Dysfunction

Sepsis is associated with the activation of coagulation pathways and endothelial dysfunction, which can lead to impaired microvascular blood flow and tissue hypoxia. This response can lead to myocardial dysfunction and ARDS.^45^

## Conclusion

The pathophysiological changes of ARDS involve pulmonary parenchyma and pulmonary circulation. Release of cytokines, activation of prothrombotic pathways, and endothelial injury along with mechanical ventilation add both chemical and physical insult to lungs and pulmonary circulation. These factors directly or indirectly involve the heart, leading to either myocardial injury with cardiac dysfunction in the form of RV failure or biventricular involvement in severe sepsis. Biomarkers of cardiac injury and cardiac stretch, troponin, and BNP are elevated in ARDS and associated with higher mortality.^46^

Similarly, certain cardiac conditions, such as cardiac arrest in postcardiotomy patients, pose a higher risk of developing ARDS. Recent literature also is discussing cardiac phenotypes in ARDS and their association with ARDS prognosis and mortality.

In patients being diagnosed with ARDS, the cardiac association cannot be ignored. All these patients should also undergo cardiac work, including cardiac enzymes, BNP, echocardiogram and, if possible, assessment of pulmonary arterial pressures along with routine ARDS workup. This will help identify underlying causes and phenotypes of ARDS, with opportunities to formulate individualized care plans within the algorithm-based ARDS and cardiac care.

## Key Points

- Acute respiratory distress syndrome (ARDs) in cardiac patients can be grouped based on cause and effect relationship.
- Heart and lung interaction leads to disease of one organ affecting the other organ based on pulmonary vascular resistance, hypoxia, and inflammation.
- ARDS has cardiac subphenotypes that can impact mortality risk.
- Hypoxemia associated with ARDS can lead to pulmonary hypertension and right ventricular dysfunction.
- Inflammatory biomarkers and infection can lead to direct myocardial injury in ARDS secondary to infectious etiology.
- Knowing the cause and effect relationship of ARDS and cardiac dysfunction can help with management.
